# Evaluating antimicrobial activity of selected medicinal plant extracts against pasteurellosis-causing bacteria in small ruminants

**DOI:** 10.3389/fvets.2025.1563208

**Published:** 2025-06-02

**Authors:** Beshada Asfa, Dereje Nigussie Woldemichael, Liyuwork Tesfaw, Lishan Asefa, Solomon Desta, Sisay Girma, Teshale Sori Tolera, Takele Beyene Tufa

**Affiliations:** ^1^School of Veterinary Medicine, Ambo University, Ambo, Ethiopia; ^2^Armauer Hansen Research Institute, Addis Ababa, Ethiopia; ^3^National Veterinary Institute, Bishoftu, Ethiopia; ^4^College of Veterinary Medicine and Agriculture, Addis Ababa University, Bishoftu, Ethiopia; ^5^School of Veterinary Medicine, Borena University, Yabello, Ethiopia; ^6^Institute for Microbiology, University of Veterinary Medicine Hannover, Hannover, Germany

**Keywords:** antibacterial activity, medicinal plants, *Mannheimia haemolytica*, *Pasteurella multocida*, pneumonic pasteurellosis, phytochemical screening, small ruminants

## Abstract

Pneumonic pasteurellosis is a highly significant infectious disease globally, posing a major economic threat to small ruminants (SR) in Sub-Saharan Africa, including Ethiopia. Although antibiotics have been effective in treating this disease, farmers in remote areas of Ethiopia often prefer traditional herbal medicines to manage respiratory infections in SR. This study aimed to evaluate the antibacterial activity of crude extracts from three selected medicinal plants—*Nicotiana tabacum, Psidium guajava*, and *Solanum incanum—*against respiratory infections caused by *Pasteurella multocida* and *Mannheimia haemolytica* in SR, in comparison to commercial gentamicin, oxytetracycline, and streptomycin antibiotics. The Agar well diffusion method was used to determine the antibacterial activity of methanol and chloroform extracts from the three medicinal plants against *P. multocida* and *M. haemolytica* strains. The phytochemical constituents of the plant extracts were analyzed by using standard phytochemical screening methods. Methanol extracts from all three plants demonstrated significant antibacterial activity against both bacterial strains (*Pasteurella multocida* and *Mannheimia haemolytica*) at a concentration of 200 mg/mL, comparable to the effectiveness of gentamicin and streptomycin. Among the three plants, *S. incanum* showed the highest zone of inhibition (26.3 mm), followed by *N. tabacum* (19.8 mm) and *P. guajava* (19.6 mm) against the tested strains. Similarly, chloroform extracts also exhibited notable activity, with *P. guajava* showing the highest inhibition (30.2 mm) against *P. multocida* at 200 mg/mL. Phytochemical screening revealed the presence of various bioactive compounds, including alkaloids, flavonoids, tannins, saponins, and terpenoids. These findings support the traditional use of these medicinal plants in treating respiratory infections caused by *P. multocida* and *M. haemolytica* in SR.

## 1 Introduction

Small ruminants (SR), particularly sheep and goats, are vital to the livelihoods of Ethiopian farmers. However, diseases contribute to significant financial losses and the socioeconomic development of rural farmers (1), due to the common occurrence of respiratory tract infections in SR (2). Pneumonic pasteurellosis is one of the most economically impactful infectious diseases affecting sheep and goats, with a global distribution. *Mannheimia haemolytica* and *Pasteurella multocida,* commensally resident in the upper respiratory tracts of healthy ruminants, are the primary etiological agents of pneumonic pasteurellosis, with aerosol transmission being the primary mode of spread. This disease causes considerable economic loss due to mortality, morbidity, decreased food availability for human consumption, reduced product quality, and the high cost of treatment (2).

Antimicrobials are the cornerstone for preventing and managing Pasteurella and Mannheimia infections. Oxytetracycline is the antibiotic of choice for treating pasteurellosis in SR, as antibiotic-resistant strains are rare in sheep and goats compared to cattle (3). Other antibiotics such as penicillin–streptomycin, tilmicosin, and florfenicol are also effective. However, indiscriminate antimicrobial use (AMU) increases the risk of selecting resistant bacteria, facilitates the spread of resistance genes, and consequently decreases the effectiveness of currently available antimicrobial agents in treating diseases of food-producing animals (4). Therefore, developing a cost-effective and socially acceptable remedy from low-cost resources to complement modern medicine is highly desirable.

In Ethiopia, farmers widely practice medicinal plant-based alternative medicines to treat both human and livestock diseases (5), with approximately 90% of livestock relying on these treatments (6). Farmers in remote areas continue to depend on traditional remedies due to limited access to pharmaceuticals and the high cost of modern drugs (7). Phytochemicals such as alkaloids, glycosides, phenolics, tannins, and saponins are biologically active compounds found in various plant parts, contributing to their medicinal properties. Pharmacologically, secondary metabolites exhibit diverse activities, including antimicrobial effects (8).

Previous research has documented medicinal plants used for treating livestock ailments (9, 10). However, few studies have focused on plants employed in the treatment of SR pasteurellosis (11, 12). Additionally, comprehensive scientific research is still lacking on the phytochemical composition and pharmacological properties of many promising therapeutic plants (13). Therefore, further investigation into the antimicrobial potential of solvent extracts from selected herbal plants traditionally used to treat pasteurellosis in SR is imperative.

*Nicotiana tabacum* (tobacco), *Psidium guajava* (guava), and *Solanum incanum* (black nightshade) have shown promising activities against respiratory infections in livestock, with guava and *Solanum incanum* exhibiting significant antibacterial effects (14, 15). A review of the literature reveals that no studies have been conducted on the absolute solvent (99.8%) extracts of *Nicotiana tabacum, Psidium guajava,* and *Solanum incanum* to assess their activities against *P. multocida* and *M. haemolytica*. This study aimed to evaluate the *in vitro* antibacterial activities of crude extracts from *N. tabacum*, *P. guajava*, and *S. incanum* against *P. multocida* and *M. haemolytica,* the bacteria responsible for pasteurellosis in SR. Additionally, the study investigated the bioactive components of these plants and compared the antibacterial activity between crude methanol and chloroform extracts.

The medicinal plants included in this study were selected based on their ethnoveterinary applications and documented antimicrobial properties (14–17). These plants are traditionally used by local farmers for treating respiratory infections in livestock in Ethiopia (14, 18). Factors such as availability, cost-effectiveness, and reported efficacy against common respiratory pathogens like *M. haemolytica* and *P. multocida* were central to their selection. By integrating traditional knowledge with scientific evaluation, this study underscores the relevance of medicinal plants in managing pneumonic pasteurellosis in SR.

## 2 Materials and methods

### 2.1 Chemicals and instruments

All chemicals, namely 99.8% chloroform (Daryaganj, New Delhi-110002, India) and 99.8% methanol (Tarapur MIDC, Boisar, Palghar, Maharashtra, India) were purchased from chemical importers at Addis Ababa, Ethiopia. Antibiotic disks of gentamicin, oxytetracycline, and streptomycin (Oxoid Ltd., Basingstoke, Hampshire, England), DMSO (Tarapur MIDC, Boisar, Palghar, Maharashtra, India), and Mueller Hinton Agar (MHA) (D-88/2, MIDC, Turbhe-400705, New Mumbai, India) were also used. Instruments, such as an electrical powder grinder, electronic analytical balance, universal bottle, filter paper, rotary evaporator, round flask, petri dish, Bunsen burner, were available in Addis Ababa University College of Veterinary Medicine and Agriculture (AAU-CVMA), National Veterinary Institute (NVI) and Ethiopian Public Health Institute (EPHI).

### 2.2 Description of the study area

The research was carried out in Bishoftu city, central Oromia region, Ethiopia. Bishoftu is located in the East Shoa Zone of the Oromia regional state. The area is located 47 km southeast of Addis Ababa and at 8°45′N, 38°59′E/8.75°N, 38.983°E with an elevation of 1920 m.a.s.l in the central highland of Ethiopia. It has an average annual rainfall of 1,150 mm, with 84% falling during the long rainy season from June to September and the remainder falling during the short rainy season from March to May. The mean annual minimum and maximum temperatures are 8.5 and 30.7°C, respectively, and the mean relative humidity is 61.3% (19).

### 2.3 Study design and sampling methods

A laboratory-based experimental study was conducted to evaluate the phytochemical properties and *in vitro* antibacterial activity of selected medicinal plants. The selection of plants was guided by a literature review of previous research findings, documented traditional uses, information gathered from local communities during plant collection, and an evaluation of the crude extract activity of medicinal plants with high use value against respiratory problems in livestock or SR. Community members reported using these medicinal plants to treat ruminants exhibiting clinical signs of coughing. Based on this information, three different plants—*Nicotiana tabacum, Psidium guajava*, and *Solanum incanum—*were chosen to investigate their pharmacological properties. The antibacterial activity of the crude extracts from these plants was assessed *in vitro* using the agar well diffusion method on reference bacterial strains (20, 21).

### 2.4 Identification of bioactive constituents

#### 2.4.1 Plant collection and preparation

In this investigation, the leaves of *N. tabacum*, *P. guajava*, and *S. incanum* plants were harvested based on different types of literature reviews and information from society. During January 2023, plant samples were collected and packed in a polyethylene bag from Bishoftu town on Babogaya Road beside the highway (approximately 8.78 latitudes, 39.00 longitudes), 50 km from Addis Ababa, Ethiopia.

Since the plants are widely grown abundantly, no access permit was required for the collection of these plants. Specimens were transported to Addis Ababa University (AAU), College of Natural Science herbarium for future reference, and taxonomically identified and authenticated by a botanist with voucher specimen numbers BA001, BA002, and BA003, respectively. After collection, plant materials were washed with running tap water to remove the soil and dust particles. Plant materials were dried under the shade for 3 weeks at room temperature and ground into a fine powder. Finally, the specimens were preserved in a closed container at 4°C for further use at AAU College of Veterinary Medicine and Agriculture (CVMA).

#### 2.4.2 Extraction of medicinal plants

The medicinal plant extraction was performed using a maceration technique (22). A 100 gram powdered sample of each plant was placed in separate round-bottomed extraction flasks and soaked in 400 mL of solvents (99.8% methanol and chloroform) at room temperature. This procedure was repeated three times, allowing the mixture to stand for 72 h with intermittent stirring to facilitate extraction. The resulting mixture was then filtered using Whatman filter paper No. 1.

To concentrate the filtrate, a rotary evaporator (Rotavapor R-200, Buchi) was used. The water bath temperature was initially set at 60°C but reduced to no more than 40°C as the extract neared dryness to yield crude residue. The crude residue was then allowed to air dry in Petri dishes before undergoing final drying in a hot-air oven at 40°C. Once dried, the concentrate was weighed, and the percentage yield calculated. The extracts were transferred into well-labeled, sterile screw-capped bottles and stored in a refrigerator at 4°C until further antibacterial testing and phytochemical screening for secondary metabolite identification. The same procedure was followed for all selected medicinal plants.

The percentage yield was determined by dividing the average weight of the extract obtained (from three replicated extraction) by the weight of the plant sample and multiplying by the hundred (23).

#### 2.4.3 Phytochemical screening

Phytochemical screening was conducted to identify various classes of compounds in the leaves of three different medicinal plants. Standard phytochemical tests were performed as previously described (24–26). The presence of alkaloids, flavonoids, phenols, saponins, steroids, tannins, and terpenoids was determined following the method suggested by Abdella et al. (26) and Sharma et al. (25).

**Alkaloids**: To 2 mL of extract, 1 mL of Mayer’s reagent (Loba Chemie Pvt. Ltd., India) was added. The formation of a brown/ reddish color precipitate indicates the presence of alkaloids.

**Flavonoid**s: 2 mL of 2% sodium hydroxide solution (Stellar Exports, India) was added to 1 mL of each extract. A yellow coloration was observed indicating the presence of flavonoids.

**Phenols**: Plant extract was dissolved in 5 mL distilled water and 3 mL of 10% lead acetate solution (Stellar Exports, India) was added. The formation of a white precipitate color indicates the presence of phenols.

**Saponins**: 2 g of powdered sample was boiled in 20 mL of distilled water. 10 mL of filtrate and 5 mL of distilled water were quivered vigorously. The appearance of frothing indicated the presence of saponin.

**Steroids**: 1 g of plant extract was dissolved in distilled water and filtered. A few drops of concentrated sulfuric acid (Loba Chemie Pvt. Ltd., India) was added. The appearance of red color in the lower layer indicates the presence of steroids.

**Tannins**: 4 mL of 10% sodium hydroxide was added to the extract. The formation of an emulsion indicates the presence of tannins.

**Terpenoids**: 0.2 g of crude extract was dissolved in 2 mL of chloroform. To this, 2 mL of concentrated sulfuric acid was added. An interface with a reddish-brown color indicates the presence of terpenoids.

### 2.5 Antimicrobial activity evaluation

#### 2.5.1 Source of bacterial strains and inoculum preparation

Two lyophilized reference bacterial strains, specifically *M. haemolytica* (serotype A_2_) and *P. multocida* (serotype A) isolated from SR, were provided by the National Veterinary Institute’s (NVI), Bishoftu, Ethiopia. In collaboration with the institute, further laboratory procedures were carried out to evaluate the *in vitro* efficacy of plant extracts.

Mueller Hinton Agar (MHA) medium was prepared by adding 38 g in 1 liter of distilled water and boiled to dissolve the powder completely. This procedure was repeated four times (total 171 g in 4.5 liter). After dissolving, sterilized with the help of an autoclave at a pressure of 151 Lbs and temperature of 121°C for 15 min. Then, all Petri dishes were filled with an equal volume of 25 mL. The bacterial strains were prepared from frozen stock, streaked on MHA plates, and incubated at 37°C for 24 h. After incubation, a single colony of each organism was picked and inoculated into 5 mL of distilled water, and the tube was shaken vigorously using a vortex shaker to obtain homogeneity of the solution. Each cultured isolate was compared with 0.5 McFarland standard, creating 10^8^ colony forming units (CFU)/ml for turbidity standards as described by Remel (27). Distilled water was used to standardize the turbidity of the isolates.

#### 2.5.2 Preparation of stock solution and serial dilution

This was carried out using the procedures outlined by Charles et al. (28). Solutions of 400 mg/mL were prepared by dissolving 800 mg of each dried crude powder in 2 mL of 5% DMSO solution. 200 mg/mL, 100 mg/mL, 50 mg/mL, 25 mg/mL, and 12.5 mg/mL working solutions of *N. tabacum*, *P. guajava*, and *S. incanum* at concentrations of were prepared and used for susceptibility testing as follows; five sterile vial bottles were arranged for each extract, and 1 mL of sterile solvent solution was dispensed into each vial. From the stock solution, 1 mL of each extract was transferred into the first vial, agitated using a vortex, and then subjected to successive two-fold serial dilutions.

#### 2.5.3 *In vitro* antibacterial activity assay

The antibacterial activities of the extracts against reference bacterial strains of *M. haemolytica* (Serotype A_2_) and *P. multocida* (Serotype A) were determined using the agar-well diffusion method described by Romha et al. (29). The inoculum of each test organism was prepared in sterile test tubes and inoculated onto the surface of MHA plates using sterile swabs, which were then evenly distributed by rocking the plate. The plates were allowed to dry at room temperature under cover to avoid atmospheric contamination.

Using a circular cork borer with 6 mm diameter, wells were created at equal distances apart in the MHA plates. On the first plate, two wells for plant extracts (at concentrations of 200 mg/mL, 100 mg/mL) and two antibiotic disks were made. On the second plate, four wells were created: three for plant extracts [at concentrations of 50 mg/mL, 25 mg/mL and 12.5 mg/mL and one for a negative control (DMSO)]. Each well was filled with 100 μL methanol and chloroform extracts of *N. tabacum*, *S. incanum*, and *P. guajava* leaves, or the negative control (DMSO). For the positive control, antibiotic disks containing Gentamicin (10 μg), Oxytetracycline (30 μg), and Streptomycin (10 μg) were carefully placed on the agar surface using forceps. The plates were pre-incubated at 4°C for 2 h to ensure uniform diffusion of the extracts into the agar, followed by incubation at 37°C for 24 h. The antibacterial activity was determined by the diffusion of the antibacterial agents in the agar medium, which inhibited the growth of the bacterial strains. Zones of inhibition (ZI) were measured in millimeters (mm) using a digital caliper. Each ZI measurement was taken twice (vertically and horizontally), and the average value was calculated to ensure accuracy. Results were recorded as the mean of three independent replicates for each extract and were compared with the effects of the antibiotic disks.

Stock and working solutions of the plant extracts were prepared in 5% DMSO diluted with distilled water. All experiments were conducted under strict aseptic conditions. According to performance standards for antimicrobial susceptibility testing, the organisms were categorized as susceptible (≥15 mm), intermediate (12–14 mm), and resistant (≤11 mm) to antibiotics based on their zone diameters (30).

### 2.6 Data analysis

The raw data were organized and stored in the Microsoft Excel database system. Statistical analysis was performed with RStudio software (version 4.4.1, 2024). To evaluate the statistical differences in the mean ZI for *P. multocida* and *M. haemolytica,* as well as the variation in bacterial susceptibility among three selected medicinal plants, a one-way analysis of variance (ANOVA) was performed. *Post Hoc* multiple comparison tests, including Tukey’s Honest Significant Difference (HSD) test, were carried out to compare parameters within and between groups at a significance level of *p* < 0.05. A paired *t*-test was used to evaluate the significance of solvents on extraction yield. All *p*-values less than this threshold were considered statistically significant. Data were presented as mean ± standard deviation.

## 3 Results

### 3.1 Yields of extracts

The percentage yield of absolute methanol and chloroform extracts obtained from *N. tabacum*, *S. incanum*, and *P. guajava* is summarized in Table 1. The highest yield was obtained from the methanol extract of *S. incanum* (13.5%), followed by *N. tabacum* (8.5%) and *P. guajava* (3.3%). In contrast, the highest yield of chloroform extracts was recorded for *N. tabacum* (4%), followed by *P. guajava* (2.1%), and *S. incanum* (1.46%). Notably, a significant difference in yield was observed between the methanol and chloroform extracts of *S. incanum.* Additionally, for *N. tabacum* and *P. guajava,* the percentage yield of methanol and chloroform extracts was approximately double that of the chloroform extracts (Table 1). These findings indicate that the methanol extracts of the three medicinal plants had a significantly higher percentage yield (*p* = 0.006) compared to their chloroform extracts.

**Table 1:** The weight in gram (g) and percentage yield of crude methanol and chloroform extracts of *N. tabacum*, *P. guajava*, and *S. incanum.*

Sample	Weight of sample in g	Methanol extract		Chloroform extract		95% CI	*p-*value
		Wt_av (g)	Yield (%)	Wt_av (g)	Yield (%)		
*N. tabacum*	300	25.5	8.5	12.2	4.0	23.87–27.13	0.0004
*P. guajava*	300	9.9	3.3	6.3	2.1	9.05–10.80	0.0045
*S. incanum*	300	40.5	13.5	4.4	1.46	37.81–43-19	0.0004

The overall *p*-value was 0.0045. Wt_av = average weight of three replicate measurements.

### 3.2 Phytochemical screening

Phytochemical analysis was done on the crude methanol and chloroform extracts to confirm the presence of bioactive components, including alkaloids, flavonoids, phenols, saponins, steroids, tannins, and terpenoids in the leaves of *N. tabacum*, *S. incanum*, and *P. guajava* (see Table 2).

**Table 2:** Qualitative phytochemical analysis of leaves extract of *N. tabacum, P. guajava*, and *S. incanum.*

Phytochemical	*N. tabacum*		*P. guajava*		*S. incanum*	
	CH₃OH	CHCl₃	CH₃OH	CHCl₃	CH₃OH	CHCl₃
Alkaloids	+	+	+	−	+	−
Flavonoids	−	+	−	+	−	−
Phenols	+	+	+	+	+	+
Saponins	+	+	+	±	+	±
Steroids	+	−	+	−	+	−
Tannin	+	+	+	+	−	+
Terpenoids	+	−	+	+	+	−

CH₃OH, Methanol; CHCl₃, Chloroform; + means presence of phytochemicals; − means absence of phytochemicals; ± found in lower.

### 3.3 Antibacterial activity study of plant extracts

The antibacterial activities of the plant extracts were evaluated using the agar well diffusion method. The findings revealed varying degrees of activity against *P. multocida* and *M. haemolytica.* The *in vitro* antibacterial activity was evaluated in the presence or absence of ZI and compared against reference antibiotics. Gentamicin, oxytetracycline, and streptomycin were selected as reference drugs due to their frequent use as first-line treatments for these bacterial infections. The observed ZI in diameter, measured from the edge of each well, differed based on increasing concentrations, solvent types, and bacterial strains. Table 3 presents the mean ZI results from triplicate experiments, spanning five different extract concentrations.

**Table 3:** Antibacterial test results of methanol and chloroform extracts *of N. tabacum, P. guajava*, and *S. incanum* with mean zone of inhibition (mm) (Mean ± Standard deviation).

Bacterial strains	Conc. (mg/ml)	Zone of inhibition (mm) (Mean ± Standard deviation)						*p*-value
		*N. tabacum*		*P. guajava*		*S. incanum*		
		Methanol	Chloroform	Methanol	Chloroform	Methanol	Chloroform	
*P. multocida* (serotype A)	200	19.8 ± 0.49	15.3 ± 0.61	19.6 ± 0.28	30.2 ± 0.41	26.3 ± 0.24	16.0 ± 0.14	0.000
	100	15.3 ± 0.57	13.3 ± 0.63	13.6 ± 0.31	28.5 ± 0.33	24.3 ± 0.22	14.6 ± 0.17	0.000
	50	12.2 ± 0.35	12.1 ± 0.2	12.6 ± 0.12	19.4 ± 0.43	20.6 ± 0.3	13.4 ± 0.14	0.000
	25	6	10.9 ± 0.27	6	15.7 ± 0.85	19.3 ± 0.49	12.0 ± 0.43	0.000
	12.5	NI	6	NI	12.9 ± 0.26	17.3 ± 0.26	10.4 ± 0.23	0.000
	10 μg CN	22.3 ± 0.37	22.3 ± 0.37	22.3 ± 0.37	22.3 ± 0.37	22.3 ± 0.37	22.3 ± 0.37	–
	30 μg OT	35.2 ± 0.5	35.2 ± 0.5	35.2 ± 0.5	35.2 ± 0.5	35.2 ± 0.5	35.2 ± 0.5	–
	10 μg S	16.6 ± 0.1	16.6 ± 0.1	16.6 ± 0.1	16.6 ± 0.1	16.6 ± 0.1	16.6 ± 0.1	–
	DMSO	NI	NI	NI	NI	NI	NI	
*M. haemolytica* (serotype A2)	200	19.0 ± 0.34	15.3 ± 0.55	18.6 ± 0.35	15.0 ± 0.5	22.2 ± 0.49	14.7 ± 0.42	0.000
	100	15.4 ± 0.62	12.2 ± 0.11	13.6 ± 0.36	13.3 ± 0.6	20.5 ± 0.19	12.8 ± 0.23	0.000
	50	13.4 ± 0.5	11.2 ± 0.14	10.3 ± 0.32	12.3 ± 0.46	18.9 ± 0.06	12.1 ± 0.31	0.000
	25	6	10.4 ± 0.17	8.7 ± 0.29	10.8 ± 0.23	16.9 ± 0.16	10.7 ± 0.05	0.000
	12.5	NI	6	6	8.6 ± 0.23	14.7 ± 0.15	9.4 ± 0.2	0.000
	CN	19.5 ± 0.55	19.5 ± 0.55	19.5 ± 0.55	19.5 ± 0.55	19.5 ± 0.55	19.5 ± 0.55	–
	OT	31.0 ± 0.5	31.0 ± 0.5	31.0 ± 0.5	31.0 ± 0.5	31.0 ± 0.5	31.0 ± 0.5	–
	S	13.0 ± 0.1	13.0 ± 0.1	13.0 ± 0.1	13.0 ± 0.1	13.0 ± 0.1	13.0 ± 0.1	–
	DMSO	NI	NI	NI	NI	NI	NI	

CN, Gentamicin; DMSO, Dimethyl sulfoxide; NI, No inhibition; OT, Oxytetracycline; S, Streptomycin.

At a concentration of 200 mg/mL, the methanol and chloroform extracts of *N. tabacum, P. guajava,* and *S. incanum* exhibited high ZI against both *P. multocida* and *M. haemolytica* serotypes. Table 3 shows that methanol and chloroform extracts of *N. tabacum* demonstrated ZI values of 19.8 mm and 15.34 mm against *P. multocida*, and 19.06 mm and 15.29 mm against *M. haemolytica*, respectively, at 200 mg/mL. These results highlight the potent antibacterial activity of *N. tabacum* extracts at this concentration. In contrast, at 12.5 mg/mL, the methanol extract of *N. tabacum* showed no inhibition, while the chloroform extract exhibited a ZI of 6 mm. On the other hand, the methanol and chloroform extracts of *P. guajava* also demonstrated notable antibacterial effects against *P. multocida*, with ZI values of 19.6 mm and 30.2 mm, respectively, at 200 mg/mL. Similarly, against *M. haemolytica*, these extracts achieved ZI values of 18.6 mm and 15 mm, respectively. In the case of *S. incanum*, the methanol and chloroform extracts yielded ZI values of 26.34 mm and 16.08 mm, respectively, against *P. multocida*, and 22.22 mm and 14.77 mm, respectively, against *M. haemolytica*, all at a concentration of 200 mg/mL.

There was a significant difference in the ZI between the three selected medicinal plant extracts at different concentrations as compared to the reference drugs. All five concentrations (200 mg/mL, 100 mg/mL, 50 mg/mL, 25 mg/mL, and 12.5 mg/mL) of chloroform extracts of the three selected medicinal plants showed significant activity against two bacterial strains when compared to the standard drugs. The highest ZI was recorded for the methanol extract of *S. incanum* (26.34 mm) and the chloroform extract of *P. guajava* (30.2 mm) on *P. multocida*. However, the methanol and chloroform extracts of *N. tabacum* and *P. guajava* demonstrated low activity at a concentration of 25 mg/mL. The variability in susceptibility of *the M. haemolytica* and *P. multocida* to the extracts was assessed using a pairwise comparison of means through ANOVA (*p* > 0.05).

There was no significant difference in susceptibility between the two bacterial strains. However, the result indicated that *P. multocida* was more susceptible to all extracts at various concentrations, as well as to the standard drugs, compared to *M. haemolytica*. The methanol extracts of *S. incanum*, *N. tabacum,* and *P. guajava* showed high ZI of 26.3 mm, 19.8 mm, and 19.6 mm, respectively, against *P. multocida*. Similarly, these extracts showed relatively strong inhibition against *M. haemolytica*, with ZI values of 22.2 mm, 19 mm, and 18.6 mm at a concentration of 200 mg/mL.

The methanol and chloroform extracts of the three plant leaves showed varying levels of ZI, some concentration showing no ZI at all. The standard antibiotic disks used in the test—gentamicin (10 μg), oxytetracycline (30 μg), and streptomycin (10 μg)—produced ZI of 22.3 mm, 35.2 mm, and 16.6 mm against *P. multocida*, and 19.5 mm, 31 mm, and 13 mm against *M. haemolytica*, respectively. Oxytetracycline exhibited the highest inhibition (53.2 mm), followed by gentamicin (22.3 mm) and streptomycin (16.6 mm).

There was no ZI observed in the negative control (DMSO). In general, it was found that bacterial growth inhibition, as indicated by ZI diameter, increased with higher concentration of plant extracts (Table 3).

## 4 Discussion

The overuse of antimicrobial agents in the treatment of infectious diseases led to numerous challenges, including the development of antimicrobial resistance (AMR) (31). As a result of the emergence of resistance, conventional antimicrobial drugs are becoming increasingly ineffective (32). Natural products contain a variety of lead compounds that could aid the discovery of novel antimicrobial agents. The secondary metabolites found in medicinal plants may combat bacteria through various mechanisms that help prevent the emergence of resistance (33).

These bioactive substances, present in different plant parts, can play complementary roles in regulating key biological processes. Their functions include immune system stimulation, modulation of gene expression in cell proliferation and apoptosis, hormone metabolism, antioxidant, antibacterial, and antiviral activities (34). Medicinal plants and herbs have been used for centuries to treat a wide range of animal and human diseases. Reports on the antimicrobial properties of medicinal plants continue to emerge globally, highlighting their potential in combating bacterial growth through mechanisms distinct from those of conventional antimicrobial agents (35).

The pharmacological activity evaluation study result of the three selected medicinal plants was conducted based on a literature review and information collected from the local community during the collection process. The findings of the present study indicate that the absolute methanol extraction yields of *N. tabacum*, *P. guajava*, and *S. incanum* were significantly higher (*p* < 0.05) compared to the yields obtained using absolute chloroform extract. The highest yield was achieved with methanol, the most polar solvent after water, while the lowest yield was observed with chloroform, a less polar solvent. This variation in extraction yields highlights the influence of solvent polarity on the extraction process, as different compounds present in the plants exhibit varying degrees of solubility. Among the three plants, the absolute methanol extract of *S. incanum* yielded the highest amount compared to the other two medicinal plants (Table 1). This suggests that the selected medicinal plants primarily contain polar bioactive ingredients rather than low-polarity bioactive compounds, reaffirming the role of solvent polarity in determining extraction efficiency. Additionally, the distribution of bioactive ingredients varies across different plant species, with some containing higher concentrations of these compounds (36).

A comparative analysis of the antibacterial activity of methanol and chloroform extracts was conducted for each medicinal plant against the tested bacterial strains. The agar well diffusion method results showed that all three plants exhibited significant antibacterial activity against the two bacterial strains, with ZI values ranging from 6 mm to 30.2 mm. The chloroform extract of *P. guajava* demonstrated the highest ZI (30.2 mm) against *P. multocida* at a concentration of 200 mg/mL, surpassing the antibacterial effectiveness of the other selected medicinal plant extracts. This finding aligns with previous studies, except for variations in concentration. When compared to reference antibiotics, the antibacterial activity of *P. guajava* was found to be comparable to oxytetracycline against *M. haemolytica*. Moreover, it exhibited superior antibacterial activity compared to gentamicin and streptomycin against both *P. multocida* and *M. haemolytica* (Table 3). These observations are consistent with previous reports on the antibacterial properties of *P. guajava,* where its chloroform extracts demonstrated more sensitivity toward the growth of *P. multocida* with MIC of 316 ± 6.2 (34).

Similarly, methanol extracts of *S. incanum* demonstrated a higher ZI at all tested concentrations compared to *N. tabacum* and *P. guajava*. It also exhibited superior activity to Gentamicin and Streptomycin against *P. multocida* and *M. haemolytica* at concentrations of 200 mg/mL and 100 mg/mL. A previous study conducted in Nigeria (37) and Ethiopia (38) reported that methanol extracts of leaves of *Solanum incanum* (*Solanaceae*) leaves possess antibacterial activity against multidrug resistance bacteria, such as *Bacillus subtilis*, *E. coli*, *S. aureus, S. pyogenes, K. pneumoniae, P. aeruginosa*, and *Salmonella typhi* isolated from human and veterinary settings. In contrast, the low antibacterial activity of methanol extracts from *N. tabacum* and *P. guajava* at 25 mg/mL, as observed in this study (Table 3), suggests that this concentration may represent the minimum inhibitory concentration (MIC) for these two bacterial strains, given their ineffectiveness at 12.5 mg/mL. The lack of activity at 12.5 mg/mL in methanol extracts and the limited antibacterial effect exhibited by chloroform extracts at the same concentration across the three medicinal plants could be attributed to the lower concentrations of bioactive phytochemicals.

The mean ZI values obtained from the present study indicate that methanol extracts of the leaves of the selected medicinal plants were more effective against *P. multocida* than *M. haemolytica* when compared to reference drugs. Remarkably, the highest activity of the three medicinal plants against the bacterial strains was observed in comparison to streptomycin, the standard reference drug. Based on these findings, it can be concluded that the crude extracts of each medicinal plant showed significant antibacterial activity at higher concentrations.

This study found that *P. multocida* was susceptible to the three selected reference antibiotics: gentamicin, oxytetracycline, and streptomycin. This findings align with previous research, which has consistently reported the susceptibility of *P. multocida* to these antibiotics (39, 40). However, some studies have reported occasional resistance to streptomycin in specific isolates (41), highlighting variability in regional resistance patterns. Similarly, *M. haemolytica* was found to be susceptible to gentamicin and oxytetracycline, in agreement with earlier findings (39, 40). However, its intermediate susceptibility to streptomycin contrasts with reports indicating higher susceptibility rates in different settings. The observed susceptibility trends in this study may reflect limited resistance development in the region, potentially due to the use of traditional remedies by farmers or restricted access to antimicrobials. The intermediate susceptibility of *M. haemolytica* to streptomycin could signal emerging resistance or strain-specific variations, emphasizing the importance of localized monitoring resistance patterns.

This study presents the results of qualitative phytochemical screening of the leaf and methanol extracts of *N. tabacum, P. guajava,* and *S. incanum*. This study highlights the presence of several bioactive compounds, such as alkaloids, saponins, tannins, terpenoids, phenolics, and steroids. Notably, flavonoids were absent in the methanol extracts of *N. tabacum* and *P. guajava*, and both flavonoids and tannins were absent in the methanol extracts of *S. incanum* (Table 2). These findings are consistent with previously reported findings (38, 42, 43), providing valuable insights into the chemical profiles of these plants.

Flavonoids were reported to be present in the methanol extract of the stem of *N. tabacum* as reported by Sharma et al. (44). Bioactive chemicals were detected in chloroform extracts in lower concentrations compared to methanol extracts, with trace amounts of secondary metabolites in each extracts. Alkaloids, steroid and terpenoids were identified in chloroform extracts of *N. tabacum* leaves, while flavonoids, phenolics, tannins and saponins were absent (45). This contrasts with our findings, which revealed the presence of alkaloids, flavonoids, phenolic, tannins and saponins in the chloroform extracts of *N. tabacum,* while steroids and terpenoids were absent. In chloroform extracts of *P. guajava,* flavonoids, phenolics, tannins, terpenoids, and saponins were detected, but alkaloids and steroids were absent. Previous phytochemical studies reported the presence of only phenolics and terpenoids in chloroform extracts of *P. guajava* (46). Similarly, phenolics, tannins, and saponins were present in the chloroform extracts of *S. incanum,* whereas alkaloids, flavonoids, steroids, tannins, and terpenoids were absent (Table 3), aligning with findings reported by Ateshim et al. (47).

Generally, this study was based on a literature review (14–18) and information gathered from society during the collection of medicinal plants. However, previous research primarily focused on the antibacterial activity of the three selected medicinal plants against bacterial diseases other than pasteurellosis in SR. Therefore, this study served as a valuable foundation for further research into the isolation and characterization of phytoconstituents from the chosen plants for potential drug development.

The current study has certain limitations. Firstly, it utilized macerated extracts of *N. tabacum*, *P. guajava,* and *S. incanum,* using only 99.8% methanol and chloroform as solvents. Due to the high cost of solvents and limited resources, other alternative extraction and fractionation techniques were not conducted. Secondly, a detailed field survey of medicinal plants commonly used by traditional healers or farmers to treat the respiratory diseases of livestock was not conducted. Thirdly, the study does not include an in-depth investigation into the pharmacological properties of the selected medicinal plants or their relationship to structural elucidation. Lastly, molecular analysis to assess the effect of these medicinal plants on the resistance and virulent genes of *P. multocida* and *M. haemolytica* were not performed.

## 5 Conclusion

This study successfully extracted bioactive compounds from the leaves of *N. tabacum*, *P. guajava*, and *S. incanum*, with polar compounds yielding higher extraction rates. Our findings demonstrate that these medicinal plants exhibit significant antibacterial activity against the two bacterial strains responsible for respiratory diseases in SR. Phytochemical screening identified several secondary metabolites—flavonoids, terpenoids, steroids, saponins, alkaloids, and tannins—known for their medicinal properties. Moreover, our results provide strong evidence that absolute methanol extracts possess a higher concentration of medicinally important secondary metabolites compared to absolute chloroform extracts. This study underscores the importance of further documentation of traditionally used medicinal plants in livestock respiratory disease management. The study also encourages future research on compound isolation and structural activity evaluation against pneumonia-causing pathogens.

## Data Availability

The raw data supporting the conclusions of this article will be made available by the authors, without undue reservation.
